# Flow-regulated lymphatic vasculature development and signaling

**DOI:** 10.1186/2045-824X-6-14

**Published:** 2014-07-09

**Authors:** Yingdi Wang, Michael Simons

**Affiliations:** 1 Yale Cardiovascular Research Center, Section of Cardiovascular Medicine, Department of Internal Medicine, 300 George St, New Haven, CT 06520, USA; 2 Department of Cell Biology, Yale University School of Medicine, New Haven, CT 06520, USA

**Keywords:** Flow, Shear stress, Signaling, Lymphatic endothelial cells, Lymphatic vascular development

## Abstract

The role of blood flow in regulating signaling pathways and gene expression in the blood vasculature is well known. Recent studies have identified equally important roles of flow-mediated signaling in the lymphatic circulation including control of lymphatic vascular growth, remodeling, regeneration and maintenance of the lymphatic fate. In this review, we summarize these advances focusing on the role of fluid dynamics in control of lymphatic vasculature formation.

## Introduction

Blood flow generates shear stress that plays critical role in regulation of vascular growth and remodeling while disturbed shear is central to a number of important vascular disease processes. Thus, physiological shear forces regulate expression of transcription factors such as c-Fos and Egr-1, growth factors (PDGF-A, PDGF-B, TGF-β) and integrins among other molecules [1-4]. On the other hand, disrupted blood flow dynamics play a key role in initiation and propagation of atherosclerosis and neointima growth [5-8]. While flow-mediated effects on vascular biology are well described little attention, until recently, has been paid to the effects of fluid flow and shear stress in the lymphatic vasculature.

Lymphatic and blood vasculatures are closely related but yet distinctly different vascular systems. The lymphatic vasculature develops from embryonic venous circulation in a series of well-defined steps that are initiated by a fate change in a subset of endothelial cells (ECs) in the cardinal vein that begin expressing lymphatic markers. These newly defined lymphatic endothelial cells (LECs) then migrate out of the cardinal vein forming jugular lymph sacs that then sprout, giving rise to the entire lymphatic vascular network [9].

Unlike the blood vasculature which is a closed system, lymphatic circulation is open with blind-ended lymphatic capillaries serving as an entry point for the interstitial fluid and immune cells that are then transported via progressively enlarging collecting vessels to the thoracic duct that then empties into subclavian vein. Furthermore, while blood flow is propelled by rhythmic contractions of the heart, mammalian lymphatic circulation is driven by a combination of hydrostatic forces and skeletal muscle contraction in the limbs augmented by opening and closing of lymphatic valves. As a result, the lymphatic flow is slower and more irregular than blood flow.

Lymphatic vessels serve a variety of roles including draining interstitial fluid and macromolecules extravasated from blood vessels and returning them to blood circulation as well as facilitating immune cell trafficking and absorption of lipids. Therefore, lymphatic circulation is important for the regulation of interstitial fluid balance, tissue homeostasis and immune surveillance. Abnormal development or malfunction of lymphatic vessels is associated with pathological conditions such as lymphedema, late-onset obesity, hypertension and cardiovascular diseases including atherosclerosis [10-15].

Although LECs, similarly to blood endothelial cells (BECs), have the ability to sense flow-mediated signaling, the role played by this signaling in lymphatic vasculature development and function is still poorly understood. Recent studies have identified contributions of lymphatic shear stress signaling to the regulation of lymphatic vascular growth, remodeling and lymphatic fate maintenance that will be the subject of this review.

### Flow-mediated signaling in lymphatic endothelial cells

In mammals, the lymphatic vasculature is composed of lymphatic capillaries (also known as initial lymphatics), pre-collectors and larger collecting lymphatic vessels. Lymphatic capillaries are blind-ended structures consisting of a single layer of overlapping ECs that lack pericyte coverage and have little or no basement membrane [9]. Similar to BECs, LECs can sense flow. When cultured in 3D collagen gels and subjected to interstitial fluid flow (flow through the junctions into the lumen), LECs show distinct morphological changes including formation of long extensions and large vacuoles. In contrast, in BECs, interstitial fluid flow causes increased networking and multicellular tubulogenesis [16]. Although LECs and BECs express some common markers, they possess distinct gene expression profiles. Interstitial flow mediated different cellular responses in BECs and LECs may attribute to different signaling pathways activated by flow in these two cell types. Interestingly, in response to shear flow (flow down the lumen of the vessels), LECs do not display changes in morphology. Instead, they align along the axis of the flow and down-regulate expression levels of pan-cadherins leading to reduced LEC barrier function [16]. Taken together, these data indicate that LECs have the ability to sense distinct signals mediated by different types of flow and to respond differentially.

LECs can also detect changes of shear stress magnitude and adjust their barrier function accordingly [17]. Increasing shear stress magnitude enhances lymphatic endothelial barrier function indicated by increased transendothelial electrical resistance (TER) [17]. Mechanistically, Rac1, a small Rho family GTPase, is involved in the signaling responsible for this process as blockade of Rac1 activity significantly reduces the enhancement of lymphatic endothelial barrier function in response to increased shear stress [17]. It is known that cAMP/PKA signaling pathway can promote enhanced endothelial barrier function, which can also activate Rac1 [18-24]. Of note, inhibition of PKA activity did not affect shear stress-mediated enhancement of lymphatic endothelial barrier function [17] suggesting that shear-induced activation of Rac1 is not PKA-dependent.

### Flow-regulated lymphatic vasculature growth

Recently, an increasing body of evidence has pointed to an important role of fluid flow in lymphatic vasculature formation. For example, it has been shown that fluid flow can work synergistically with growth factors such as VEGF to promote lymphangiogenesis [25]. In a 3D fibrin gel system, in which fibrin gel is mixed with a fibrin-bound form of VEGF (α2-PI_1–8_-VEGF_121_) and LECs which then are pipetted into an interstitial flow culture chamber; interstitial flow enhances the effects of the fibrin-bound VEGF (α2-PI_1–8_-VEGF_121_) in promoting lymphatic network formation [25]. Notably, in this system, lymphatic structures are formed in parallel to flow only under the condition of the combined fibrin-bound VEGF (α2-PI_1–8_-VEGF_121_) and interstitial flow [25]. This result is correlated with interstitial flow induced changes of the extracellular distribution of soluble matrix metalloproteinases (MMPs) that release fibrin-bound VEGF (α2-PI_1–8_-VEGF_121_) to form a VEGF gradient [25].

The role of interstitial fluid flow in lymphangiogenesis has been investigated in vivo using a mouse skin regeneration model [26,27]. In this model, a circumferential section of tail skin is removed midway up the tail resulting in completely interrupted lymphatic vascular network whereas big blood vessels remain intact. A diameter-matched silicone sleeve was placed over the wound and fixed in place to the intact skin on the proximal and distal edges. 0.3% type I rat tail collagen solution was injected into the wound under the sleeve and allowed to gel; this formed a collagen dermal equivalent (CDE) [26]. During tissue regeneration, interstitial fluid channels are formed in CDE prior to the formation of lymphatic vessels, which bridge the distal and proximal portions of the lymphatic vessels in intact tail skin. Once formed, the channels guide LEC migration and formation of a functional lymphatic capillary network. Importantly, these processes take place only in the direction of interstitial flow [26].

In line with these results, reduced interstitial flow during tissue regeneration results in decreased LEC migration and lymphatic vasculature formation. VEGF-C administration fails to induce lymphangiogenesis in this setting [27]. During skin regeneration, MMPs activity and VEGF-C expression are more pronounced at the upstream than the downstream of interstitial flow [26]. These data suggest that MMPs and VEGF-C transport are likely directed by interstitial fluid flow [26]. Consistent with this hypothesis, reduced interstitial flow results in significantly increased sub-dermal MMPs and VEGF-C accumulation [27]. Together, these data suggest that growth factors and proteases transported by interstitial flow may be important for lymphatic vasculature formation [27].

Interestingly, in the tail skin regeneration model, BEC migration and vascular network organization in CDE are not directional [26] indicating that LECs and BECs have different genetic programs in response to interstitial fluid flow.

Interstitial fluid also generates pressure, which has recently been shown to be involved in lymphangiogenesis in mice. In developing mouse embryos, the volume of fluid in the interstitium is correlated with LEC morphologic changes, proliferation and lymphatic vessel expansion [28]. In response to increased interstitial fluid, LECs become elongated, show increased proliferation and enhanced VEGFR3 tyrosine phosphorylation. In contrast, a reduction in the amount of interstitial fluid causes the opposite effect – reduced LEC proliferation and decreased VEGFR3 phosphorylation [28]. Although LECs become elongated in response to changes in the volume of interstitial fluid, this elongation requires a molecular mediator that converts the fluid-induced mechanical forces to the observed cellular responses. It appears that β1 integrins play the role of “mediator” in this process [28]. When the amount of interstitial fluid is increased, β1 integrins are activated, which subsequently activates Src Family Kinases (SFKs) and the activation of SFKs results in tyrosine phosphorylation of VEGFR3 [28].

### Flow in lymphatic valve development

A recent study has identified lymph flow as a factor that regulates lymphatic valve development during lymphatic remodeling [29]. Lymphatic valves form in collecting lymphatic vessels, thereby preventing back flow of lymph. This is a complex process that involves a large number of genes and a variety of intracellular signaling pathways (reviewed by [30] which include ephrin-B2, Foxc2, Prox1, connexins, Ang2, and integrin-α9/FN-EIIIA, Sema3A/NRP1 and calcineurin/NFATc1 signaling [29,31-39].

**Figure 1:**
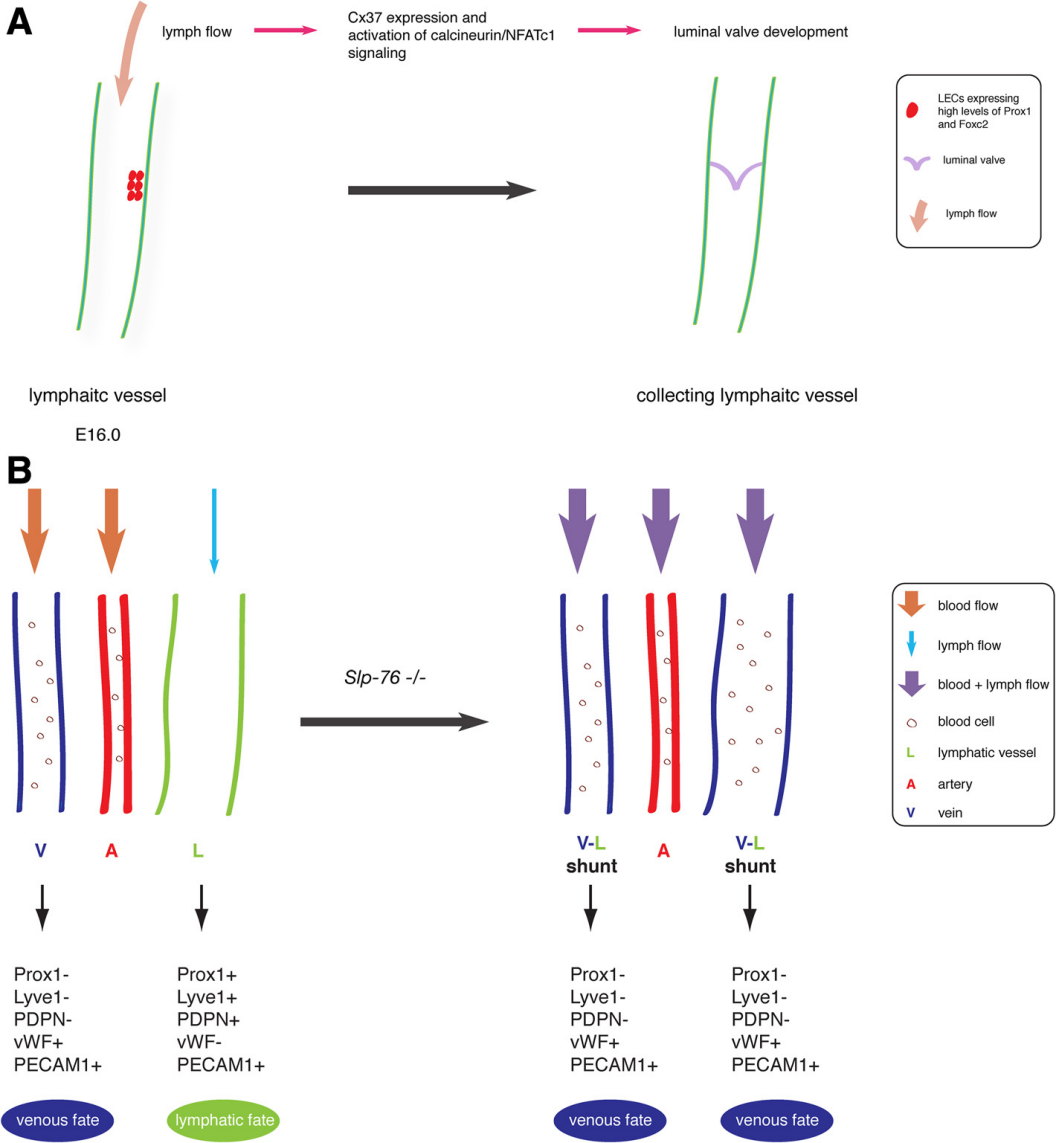
**Fluid flow in lymphatic vasculature development in mice. (A)** Flow triggers lymphatic valve development. At approximately embryonic day (E)16.0, lymph flow induces Cx37 expression and activates calcineurin/NFATc1 signaling in lymphatic-valve-forming cells initiating luminal valve development. **(B)** Fluid flow plays a role in lymphatic fate maintenance. In mice, loss of *Slp-76* results in shunt formation between blood and lymphatic vessels in mesentery and, therefore mixing of blood and lymphatic circulation. The presence of blood flow in lymphatic vessels in *Slp-76* null mice causes the change of lymphatic fate to a venous identity.

In mice, lymph flow in mesenteric lymphatic vessels commences on embryonic day (E) 15.5 and at this time lymphatic valves have not yet formed [29]. As a result, reverse lymph flow does occur in many places, especially at vessel bifurcations. This flow dynamics induce lymphatic valve development at ~ E16.0. At that time, lymphatic-valve-forming cells (LVCs) begin to express high levels of Prox1 and Foxc2, followed by the induction of Cx37 and activation of calcineurin/NFATc1 signaling [29] (Figure 1A). In cultured LECs, oscillatory fluid shear stress (OSS) that mimics lymph flow reversal occurring prior to lymphatic valve development, is able to trigger Cx37 expression and activate calcineurin/NFATc1 signaling as indicated by the nuclear accumulation of the transcription factor NFATc1 [29]. This process is also dependent upon the expression levels of PROX1 and FOXC2.

Upon exposure to OSS, LECs with high levels of PROX1 and FOXC2 accumulate NFATc1 in the nucleus, which does not occur in LECs expressing low levels of PROX1 and FOXC2. These data suggest that flow-induced shear stress activates distinct signaling pathways in different LEC populations [29]. Together, both the in vivo and in vitro data support the notion that lymph flow is involved in triggering lymphatic valve formation.

Furthermore, it now appears that different types of flow shear stress result in distinct cell morphology and actin cytoskeleton organization changes in LECs [29]. When subjected to OSS, LECs adapt a more cuboidal shape and have increased amounts of short perinuclear F-actin stress fibers, which are hallmarks of LVCs in vivo. Upon laminar shear stress (LSS), LECs become elongated, align in the direction of flow and form stress fibers, reminiscent of LECs (lymphangion cells) that express low levels of Prox1 and Foxc2 and are located adjacent to LVCs [29].

Similar to lymphatic vessels, veins also have luminal valves. Interestingly, both venous and lymphatic valve ECs share common molecular regulators [40]. For instance, molecules such as Prox1, ephrin-B2, Vegfr3 and integrin-α9 that are important for lymphatic valve development are also expressed in venous valve ECs [40]. These data suggest common signaling pathways are involved in venous and lymphatic valve formation. Fluid flow mediates distinct shear forces in veins and in lymphatic vessels. It remains intriguing to investigate the role of flow in venous valve development and how different shear forces regulate luminal valve formation in veins and lymphatic vessels through common molecular regulators.

### Fluid flow and lymphatic fate maintenance

Fluid flow is also important in the lymphatic fate maintenance. SLP-76 is a hematopoietic intracellular signaling protein, which has been shown to be required for blood and lymphatic vascular separation [41-43]. In mice, loss of *Slp-76* results in the formation of abnormal connections between blood and lymphatic vessels, and thus mixing of blood and lymphatic circulation [41-43]. Moreover, mesenteric lymphatic vessels in *Slp-76* null mice lose their lymphatic fate and adapt a venous identity [43] (Figure 1B). This phenotype has been attributed to the presence of blood flow in mesenteric lymphatic vessels in SLP-76 deficient mice and it is not due to a response to factors in the blood that are not present in the lymph [43]. These findings highlight the importance of hemodynamic forces in the maintenance of LEC identity even though the molecular mechanisms underlying this process remain unknown.

## Conclusions

Recent research advances have largely improved our knowledge on gene products and signaling pathways involved in lymphatic vascular development and have expanded the list of players that contribute to this process. An increasing amount of evidence has elevated the importance of fluid flow in the formation of lymphatic vasculature. Although our understanding of the molecular mechanism(s) underlying flow-regulated lymphatic vascular development is very limited, the importance of fluid flow in this process will draw increasing research efforts and will help to gain further insight into this fascinating research area.

## Competing interests

The authors declare that they have no competing interests.

## Authors’ contribution

YW and MS prepared and wrote the manuscript. All authors read and approved the final manuscript.
